# A VP1 mutation acquired during an enterovirus 71 disseminated infection confers heparan sulfate binding ability and modulates *ex vivo* tropism

**DOI:** 10.1371/journal.ppat.1007190

**Published:** 2018-08-03

**Authors:** Eirini D. Tseligka, Komla Sobo, Luc Stoppini, Valeria Cagno, Fabien Abdul, Isabelle Piuz, Pascal Meylan, Song Huang, Samuel Constant, Caroline Tapparel

**Affiliations:** 1 Department of Microbiology and Molecular Medicine, University of Geneva Medical School, Geneva, Switzerland; 2 Tissue Engineering Laboratory, HES-SO/University of Applied Sciences, Geneva, Western Switzerland; 3 Institute of Microbiology, Centre Hospitalier Universitaire Vaudois, Lausanne, Switzerland; 4 Epithelix Sàrl, Geneva, Switzerland; Chang Gung University, TAIWAN

## Abstract

Enterovirus 71 (EV71) causes hand, foot and mouth disease, a mild and self-limited illness that is sometimes associated with severe neurological complications. EV71 neurotropic determinants remain ill-defined to date. We previously identified a mutation in the VP1 capsid protein (L97R) that was acquired over the course of a disseminated infection in an immunocompromised host. The mutation was absent in the respiratory tract but was present in the gut (as a mixed population) and in blood and cerebrospinal fluid (as a dominant species). In this study, we demonstrated that this mutation does not alter the dependence of EV71 on the human scavenger receptor class B2 (SCARB2), while it enables the virus to bind to the heparan sulfate (HS) attachment receptor and modifies viral tropism in cell lines and in respiratory, intestinal and neural tissues. Variants with VP1_97L_ or VP1_97R_ were able to replicate to high levels in intestinal and neural tissues and, to a lesser extent, in respiratory tissues, but their preferred entry site (from the luminal or basal tissue side) differed in respiratory and intestinal tissues and correlated with HS expression levels. These data account for the viral populations sequenced from the patient’s respiratory and intestinal samples and suggest that improved dissemination, resulting from an acquired ability to bind HS, rather than specific neurotropism determinants, enabled the virus to reach and infect the central nervous system. Finally, we showed that iota-carrageenan, a highly sulfated polysaccharide, efficiently blocks the replication of HS-dependent variants in cells and 2D neural cultures. Overall, the results of this study emphasize the importance of HS binding in EV71 pathogenesis and open new avenues for the development of antiviral molecules that may prevent this virus’s dissemination.

## Author summary

Enterovirus 71 (EV71) has been the cause of major hand-foot-and-mouth disease outbreaks, particularly in the Asia-Pacific region. However, the recent emergence of severe neurological cases associated with EV71 infection in Europe and the lack of an efficient vaccine or antiviral agent to fight EV71 infections highlight two critical needs: (A) the identification of ill-defined viral factors that contribute to viral dissemination and pathogenesis in humans and (B) the development of effective antiviral strategies. Herein, based on clinical observation in an immunocompromised host, we have demonstrated that heparan sulfate attachment receptor played a critical role in EV71 virulence and that “in host” EV71 adaptation to a heparan sulfate-dependent virus was likely responsible for its dissemination. To our knowledge, this is the first study highlighting the key determinants of EV71 dissemination based on a clinical case and proposing a new therapeutic approach against EV71 neurological diseases.

## Introduction

Enterovirus 71 (EV71) is one of a limited number of enterovirus genotypes that have the ability to infect the central nervous system (CNS) [1–3] and has emerged as a major health-care threat across the Asia-Pacific region [4–7]. Although this virus is typically associated with mild hand, foot and mouth disease (HFMD) epidemics, EV71 has been increasingly associated with neurological disorders that range from aseptic meningitis with or without pulmonary edema to brain stem encephalitis and poliomyelitis-like acute flaccid paralysis, particularly among children less than 6 years old [8–10]. EV71 transmission depends on hygiene, water quality, and the extent of crowding [11]. EV71 is typically transmitted through fecal-oral routes, although transmission via respiratory secretions also frequently occurs, particularly in countries with high standard sanitation [12, 13].

Following infection, EV71 predominantly replicates in the intestinal mucosa and, to a lesser extent, in the respiratory mucosa [5, 14]. Subsequently, the virus can enter the bloodstream and disseminate to a variety of organs, including the central nervous system (CNS) [15]. EV71 dissemination to the CNS remains a rare event and the viral neurotropic determinants remain ill-defined, despite extensive epidemiological studies and experimentation in animal models.

EV71 tissue tropism may be modulated by its ability to bind to a variety of receptors [16]. The human scavenger receptor class B2 (SCARB2), a major transmembrane lysosomal protein, is ubiquitously expressed and is the best studied receptor [17]. In the human CNS, SCARB2 is expressed on neurons and glial cells [18], and a transgenic mouse model expressing human SCARB2 has exhibited susceptibility to EV71 infection and the development of ataxia, paralysis and death [19]. The second EV71 entry receptor, P-selectin glycoprotein ligand-1 (PSGL-1), is expressed exclusively on leukocytes and is only bound by certain EV71 strains [20, 21]. Finally, attachment molecules, such as heparan sulfate glycosaminoglycan (HS) [22], sialic acids [23], nucleolin [24], vimentin [25] and annexin II [26] also enhance EV71 infectivity and may contribute to viral dissemination and neurotropism. Residues that are crucial for the binding of EV71 to certain of these receptors have been mapped on the VP1 viral capsid protein. For instance, 172Q and adjacent amino acids in the EF loop are required for binding to human SCARB2 and for efficient infection of cells expressing this receptor [27] while K98E, E145A and L169F substitutions confer binding ability to murine SCARB2 [28]. Similarly, EV71 strains with a glycine or a glutamine at position 145 of VP1 are able to bind PSGL-1, while strains with a glutamate at this position are not [21]. Binding of EV71 to annexin II has been mapped to the BC loop region of VP1 (residues 40–100) [26]. Finally, positively charged residues at the 5-fold axis of EV71 capsids (VP1 162K, 242K, and 244K) appear to be essential for attachment to HS. When these residues are mutated, compensatory mutations (E98A, T100K and Q145R-T237N) can restore binding. Similarly, strains with 98E and 145E are poor HS binders, while E98K restores binding [29]. These observations are based on *in vitro* adapted EV71 isolates and do not address the *in vivo* significance of dissemination and neurotropism. In addition, the implication of immune escape mutations may add a level of complexity. VP1 residues 98 and 145 have been shown to be under positive selection and may be targeted by neutralizing antibodies [30, 31]. Finally, EV71 virulence determinants that have been identified in animal models contradict epidemiological studies in humans. VP1 residue 145E was shown to be a virulence factor for both cynomolgus monkeys [31, 32] and mice [33, 34], while isolates with 145G/Q are also frequently associated with severe neurological disease in humans [35–38]. Mutations in other genomic regions may explain these apparent contradictions.

We previously isolated EV71 from an immunocompromised patient with disseminated disease. Genomic analysis revealed that this clinical strain clusters with EV71 in the human EV-A species and is related to the subgenogroup C1. Comparison of five full-length genomes sequenced directly from respiratory, gastrointestinal, nervous system and blood specimens highlighted a critical non-synonymous single amino acid change within the EV71 VP1 BC loop (L97R) that could potentially lead to dissemination in natural infections. EV71-VP1_97R_, which was absent in the respiratory sample, was present as a dominant population in blood and cerebrospinal fluid samples and as a mixed population (EV71-VP1_97R/L_) in the stool sample [39]. However, the mechanisms by which the mutant virus could disseminate are not clear. In this study, we investigated the capacity of EV71-VP1_97L_ and EV71-VP1_97R_ to bind and mediate infection in different cell lines and in 3D reconstituted respiratory, intestinal and neural tissues. We demonstrated that both derivatives are dependent on SCARB2 for effective infection, while their specific tropism is linked to a different ability to bind HS and further correlates with the expression level of this attachment receptor in the tested tissues. This study provides new insight regarding HS binding as a critical determinant of EV71 dissemination in humans and opens the door to antiviral strategies aimed at preventing EV71 in-host adaptation and dissemination.

## Results

### EV71-VP1_97R_ and EV71-VP1_97L_ variants exhibit different cell tropism

We previously showed that the VP1 L97R mutation conferred a binding advantage in RD and Vero cells. In addition, we showed *in vitro* that this mutation was associated with a second non-conservative mutation (E167G/A) in the VP1 EF loop [39]. To define the role of these mutations more precisely, we generated 4 infectious clones with distinct combinations of each of the mutations (Table 1).

**Table 1:** Schematic representation of the 4 infectious clone constructs and spontaneous mutations arising in culture.

Construct name	VP1 changes in construct	Spontaneous mutations
*EV71-VP1_97L167E_	L^0^_97_ E^−^_167_	No
**EV71-VP1_97L167E_**	L^0^_97_ G^0^_167_	97 L to R
**EV71-VP1_97R167E_	R^+^_97_ E^−^_167_	167 E to G/A
**EV71-VP1_97R167G_**	R^+^_97_ G^0^_167_	No

*Virus sequenced from the bronchoalveolar lavage of a patient with disseminated EV71 infection.

**Virus sequenced from the stool, blood and cerebrospinal fluid of a patient with disseminated EV71 infection [39]. +, positive charge; -, negative charge; o, neutral charge.

**Fig 1:**
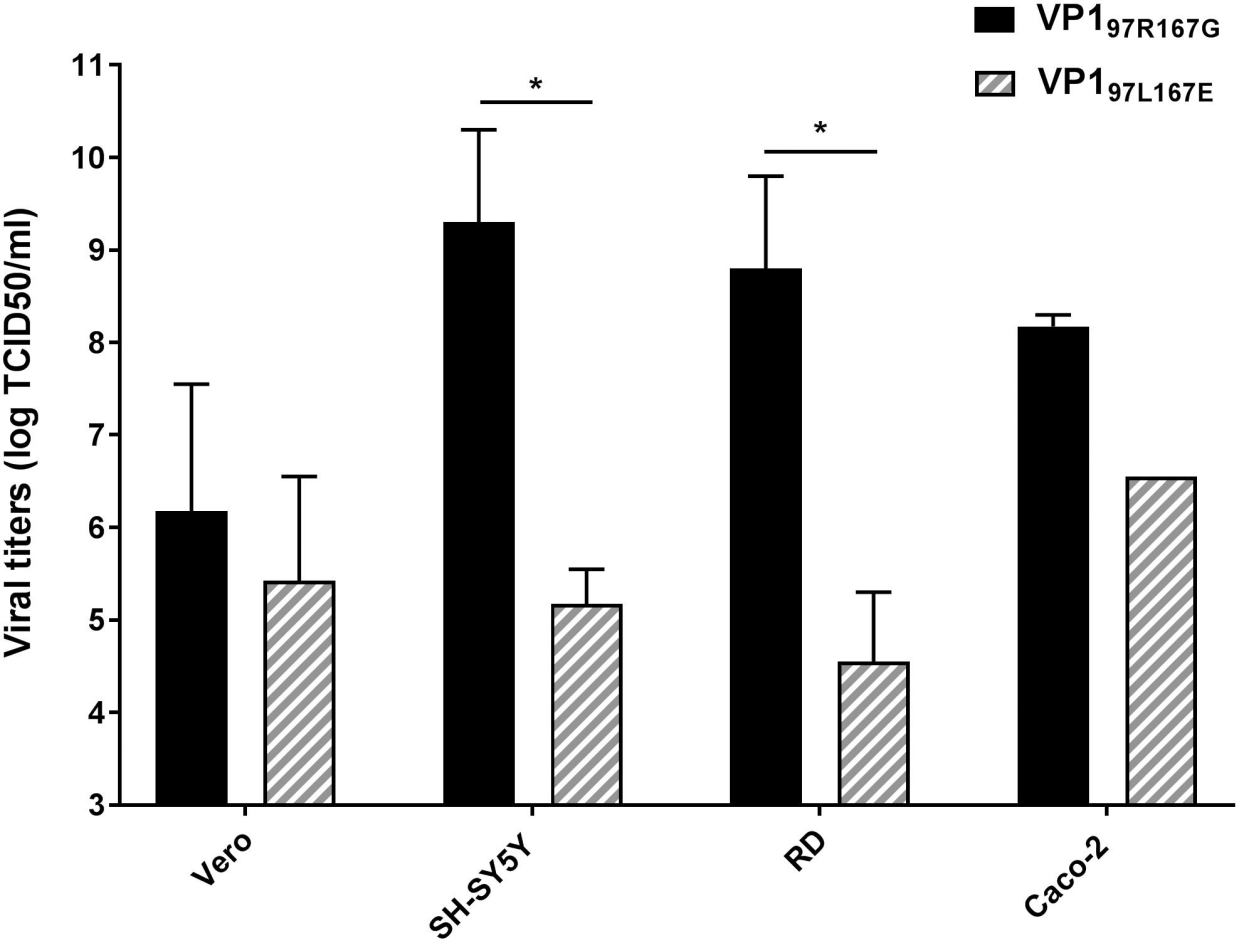
Differential cell tropism EV71-VP1_97R167G_ and EV71-VP1_97L167E_. The mean (N = 2) viral titers (±SEM) expressed as log TCID_50_/ml (median tissue culture infective dose) obtained 4 dpi are indicated. *P<0.05.

Viral stocks were prepared in monkey kidney cells (Vero) and titrated in Vero, colorectal carcinoma (Caco-2), rhabdomyosarcoma (RD) and neuroblastoma cells (SH-SY5Y). After 4 days of infection, viruses were sequenced from each cell line. EV71-VP1_97L167G_ and EV71-VP1_97R167E_ viral stocks were unstable independent on the cell line (Table 1) and thus could not be amplified and purified in sufficient quantities to be used in functional assays. EV71-VP1_97L167E_ (originally isolated from the lower respiratory tract sample of the patient with a disseminated infection) and EV71-VP1_97R167G_ (*in vitro* adapted from EV71-VP1_97R167E_, which was originally isolated from the stool, cerebrospinal fluid and blood of the patient) were thus used for all further investigations. Titration showed that EV71-VP1_97L167E_ and EV71-VP1_97R167G_ exhibited differential cell tropism, with significantly enhanced replication being observed for EV71-VP1_97R167G_ in RD and SH-SY5Y cells (Fig 1). Notably, the RNA load measured in the stocks prepared in Vero cells was comparable for the two viruses (12.38 log RNA copies/ml for EV71-VP1_97R167G_ and 12.30 log RNA copies/ml for EV71-VP1_97L167E_).

### EV71-VP1_97R167G_ and EV71-VP1_97L167E_ infections are dependent on SCARB2

**Fig 2:**
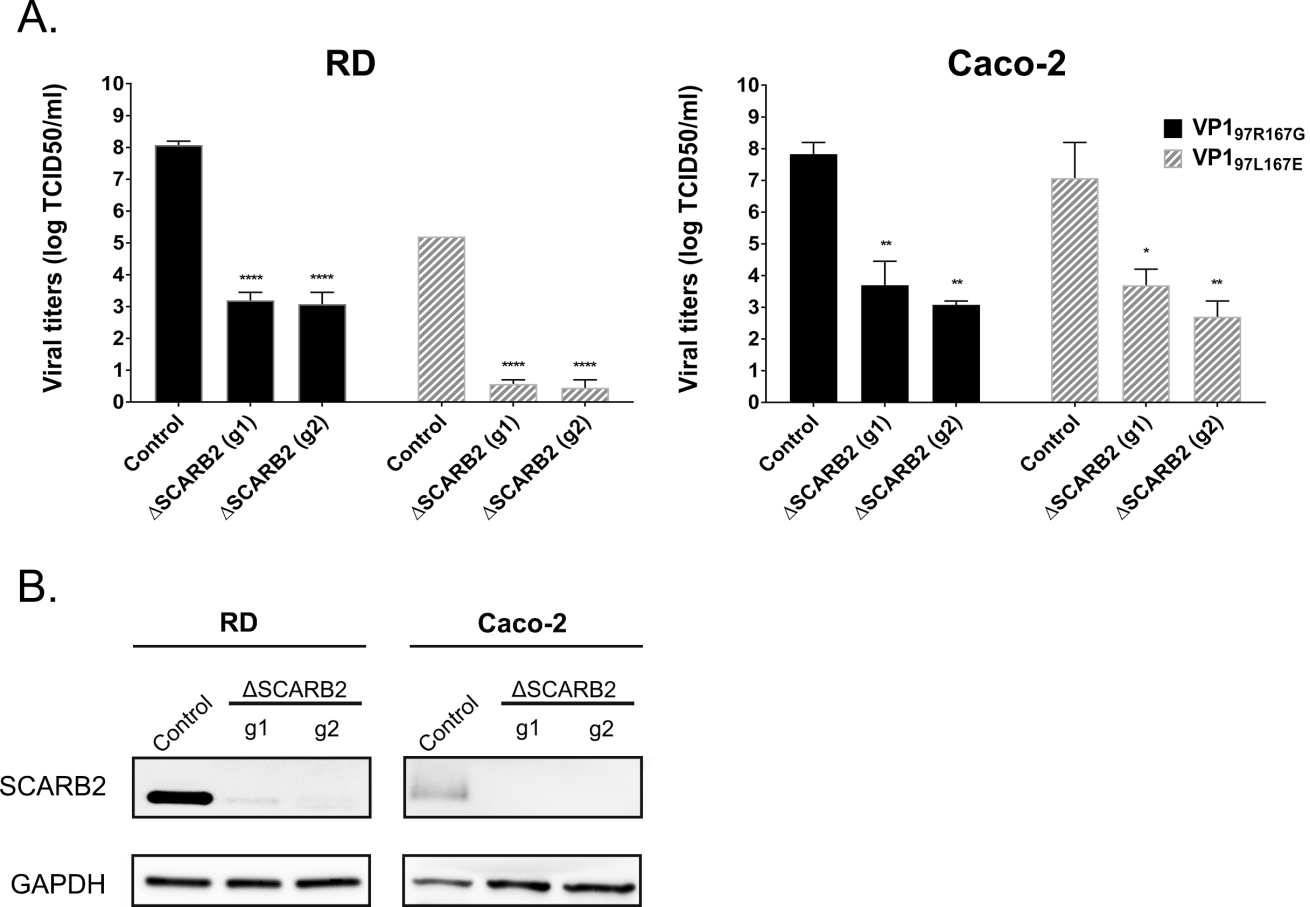
SCARB2 is necessary for effective infection by EV71-VP1_97R167G_ and EV71-VP1_97L167E_. **A)** Mean (N = 2) viral titers (±SEM) expressed as log TCID_50_/ml (median tissue culture infective dose) obtained 5 dpi in cells expressing (control) or not (ΔSCARB2) SCARB2. Wells with less than 50 infected cells were considered as negative. **B)** Western blot showing SCARB2 expression in ΔSCARB2 cells and control cells. ****P<0.0001, **P<0.01, *P<0.05.

As SCARB2 plays a critical role for EV71 infection [17], we investigated whether the differential cell tropism of the two variants is linked to a different dependence on this receptor. We used the CRISPR/Cas9 system to knockout SCARB2 in Caco-2 and RD cells, two cell lines presenting different susceptibilities to the two variants (Fig 1). A striking reduction in viral titers was observed in SCARB2 knockout RD and Caco-2 cells for EV71-VP1_97L167E_ and EV71-VP1_97R167G_ (Fig 2) suggesting that both variants depend on this receptor for effective infection regardless of the cell line. This was also shown by transfection of human SCARB2 in mouse L929 cells that are non-permissive for infection by EV71 [17]. Exogenous expression of human SCARB2 conferred susceptibility to both EV71-VP1_97L167E_ and EV71-VP1_97R167G_ (S1 Fig) confirming that the two viruses are dependent on SCARB2.

### Differential cell tropism of EV71-VP1_97R167G_ and EV71-VP1_97L167E_ is related to differential use of the heparan sulfate attachment receptor

**Fig 3:**
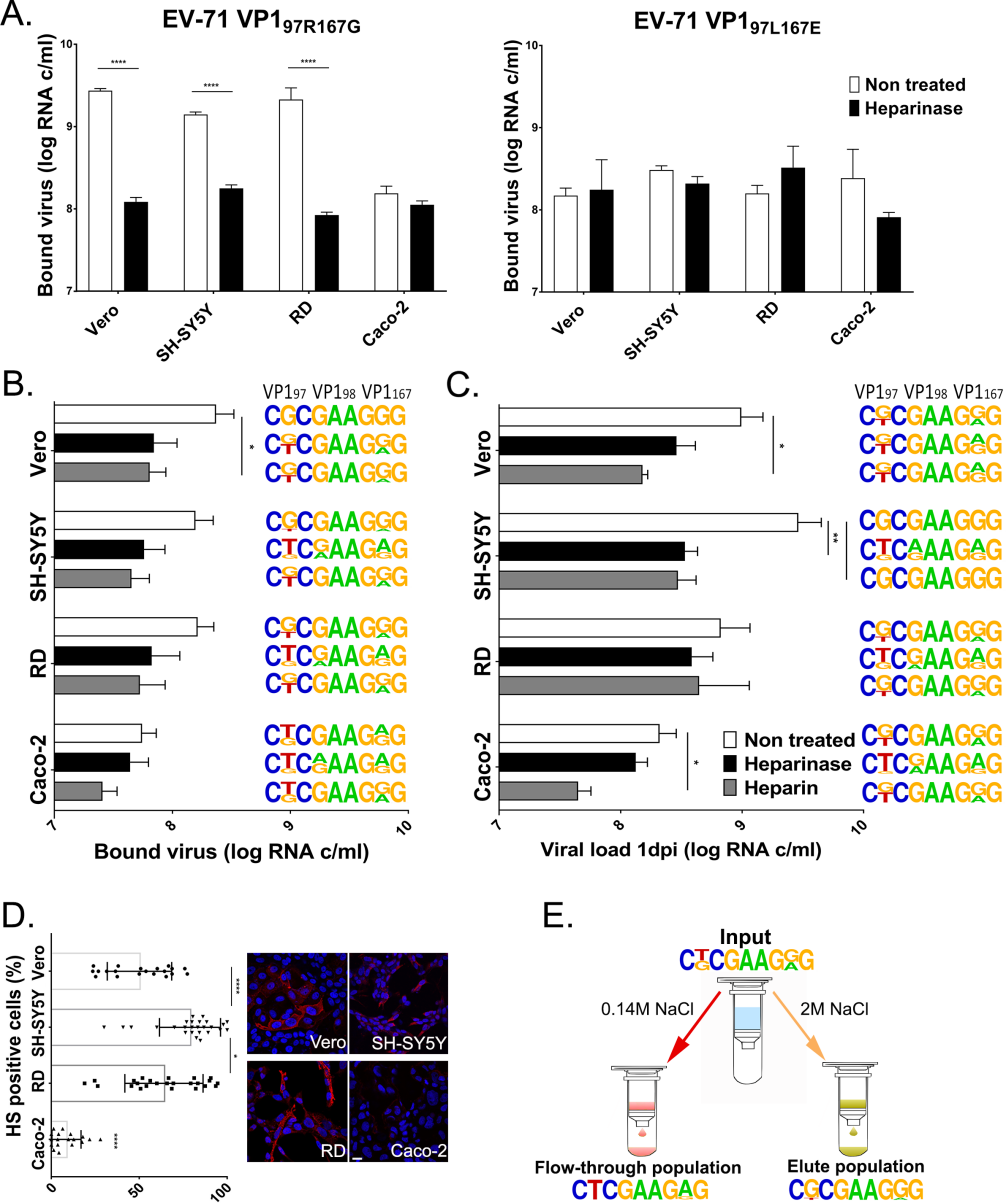
Differential cell tropism of EV71-VP1_97R167G_ and EV71-VP1_97L167E_ is linked to HS binding and correlates with its expression level at the cell surface. **(A).** Binding of EV71-VP1_97R167G_ (left panel) and EV71-VP1_97L167E_ (right panel) in the presence or absence of HS. Binding **(B)** and replication **(C)** of a viral stock composed of equivalent amounts of EV71-VP1_97R167G_ and EV71-VP1_97L167E_ in different cell lines and under conditions preventing attachment to HS. Viral loads were measured by RT-qPCR (real-time quantitative reverse transcription PCR) in whole cell extracts for binding assay and both cell extracts and supernatants for replication assay, and expressed as the mean (±SEM, N≥2) relative to the EV71-VP1_97R167G_ load in non-treated RD cells (100%). **(D).** Quantification of HS-expressing (positive) cells was achieved through an analysis using MetaMorph software (left panel) based on the immunofluorescence labeling (right panel) of HS attachment receptors at the surface of Vero, RD, SH-SY5Y cells and Caco-2 cells. More than 500 cells were included in the analysis. Scale bar = 20 μm. **(E)** Binding of EV71-VP1_97R/L167G/E_ to heparin sepharose beads. Viral population present in the input, flow-through and elute population was characterized by sequencing. ****P<0.0001, ***P<0.001, **P<0.01, *P<0.05. For B, C and E, the frequency plot [http://weblogo.berkeley.edu/] of codons encoding VP1 aa 97, 98 and 167 are shown and are representative of biological replicates (N≥2). Arginine (R) at position 97 is encoded by CGC, Leucine (L) by CTC; Glutamate (E) at position 98 is encoded by GAA, Lysine (K) by AAA; Glycine (G) at position 167 is encoded by GGG, Glutamate (E) by CTC.

We then investigated the implication of another ubiquitously expressed receptor, the HS attachment receptor, in this observed differential cell tropism. We measured the amount of EV71-VP1_97R167G_ and EV71-VP1_97L167E_ viruses bound to untreated cells or cells pretreated with heparinase III (Fig 3A). EV71-VP1_97R167G_ exhibited a binding advantage over EV71-VP1_97L167E_ in RD, Vero and SH-SY5Y cells but not in Caco-2 cells. This advantage was suppressed after cleavage of HS from the cell surface (Fig 3A, left panel). In contrast, EV71-VP1_97L167E_ did not show a significant difference in any cell line (Fig 3A, right panel).

To confirm this observation, competition experiments were performed with a viral stock (EV71-VP1_97R/L-167G/E_) containing equivalent amounts of each derivative. The competition was run under 3 conditions: using untreated cells, using cells having surface HS digested with heparinase, or using a viral population that was pretreated with heparin, a soluble HS analogue. Both binding (1 hour post-infection [hpi]) and replication (24 hpi) efficiencies were monitored by real time-quantitative polymerase chain reaction (RT-qPCR), and the dominant species was characterized by Sanger sequencing for each binding (Fig 3B) and replication condition (Fig 3C). The overall binding was lower for the mixed population than for the EV71-VP1_97R167G_ stock (Fig 3B versus 3A). Accordingly, the pretreatment of cells with heparinase reduced viral binding less significantly, while viral replication was affected in SH-SY5Y, Vero and Caco-2 cells. Sanger sequencing of the entire VP1 region after binding showed that in Vero, SH-SY5Y and RD cells, EV71-VP1_97R167G_ (with R at position 97 encoded by CGC and G at position 167 encoded by GGG) was the dominant variant in non-treated conditions, which was not the case in Caco-2 cells, where EV71-VP1_97L167E_ (with L at position 97 encoded by CTC and E at position 167 encoded by GAG) and EV71-VP1_97L167G_ were dominant (Fig 3C). Upon heparinase treatment, the dominant consensus sequence shifted towards EV71-VP1_97R/L167G/E_, EV71-VP1_97L167G/E_ or EV71-VP1_97L167E_ in binding and replication assays. Notably, viral species with a positive charge at VP1 position 98 (K encoded by GAA instead of E encoded by AAA) emerged after treatment of SH-SY5Y, RD and Caco-2 cells with heparinase (Fig 3C and 3D). Pre-incubation of viruses with heparin also resulted, although in a less stringent manner, in a shift of the dominant population from EV71-VP1_97R167G_ to a mixed EV71-VP1_97R/L167G/E_ population, except after replication in SH-SY5Y and RD cells.

To determine if the improved binding and replication of EV71-VP1_97R167G_ in cells correlated with HS expression levels, quantification of HS expression was assessed by immunofluorescence (Fig 3D and 3E). SH-SY5Y, RD and Vero cell lines strongly express HS, while Caco-2 showed low HS expression.

A recent publication showed that variants with VP1 E167G do not bind heparin [29], and the differential binding and replication of EV71-VP1_97R167G_ confirmed our previous observations with EV71-VP1_97R167E_ in SH-SY5Y and Vero cells [39], indicating that the acquired HS binding ability is due only to the L97R substitution.

Finally, we confirmed the different affinity of EV71-VP1_97R167G_ and EV71-VP1_97L167E_ for HS using a heparin sepharose binding assay. After incubation of a mixed viral stock (EV71-VP1_97R/L-167G/E_) composed of 50% of each derivative with sepharose beads, we sequenced the viral populations present in the non-heparin-binding population (flow-through) or in the heparin-binding population (eluate) (Fig 3E). As expected, EV71-VP1_97L167E_ was highlighted in the non-heparin-binding population, while EV71-VP1_97R167G_ was detected in the heparin-binding population.

### Differential tissue tropism of EV71-VP1_97R167G_ and EV71-VP1_97L167E_ variants is related to differential use of the heparan sulfate attachment receptor

To better replicate the viral tropism observed in the patient, infection assays were performed with EV71-VP1_97R167G_, EV71-VP1_97L167E_ and EV71-VP1_97R/L167G/E_ in complex tissue models, *in vitro* reconstituted from human primary cells, that mimic the upper respiratory tract, small intestine and neural tissues composed of neurons and glial cells.

#### Respiratory tissues

**Fig 4:**
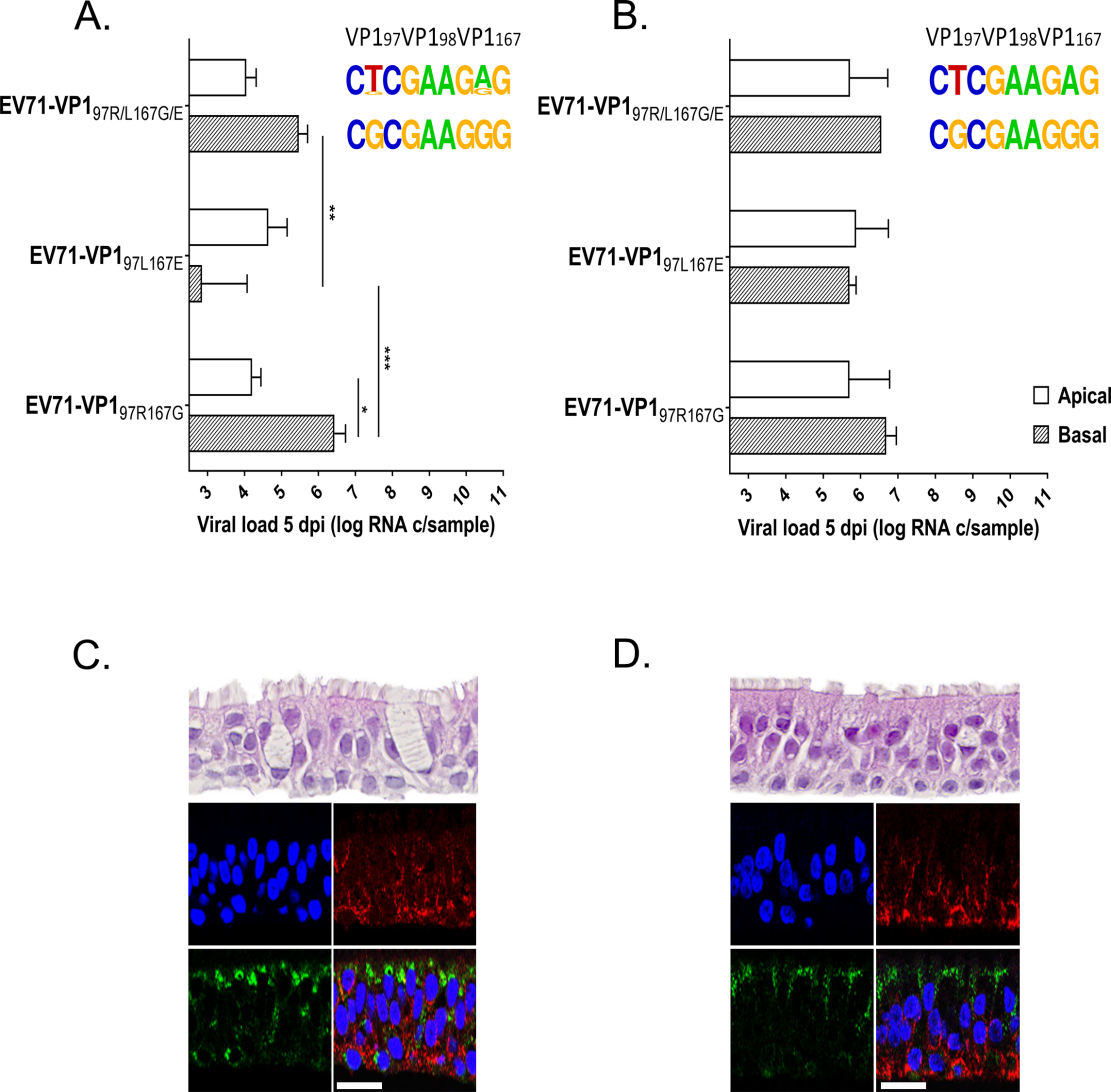
**Replication of EV71-VP1**_**97R167G**_**, EV71-VP1**_**97L167E**_ **and EV71-VP1**_**97R/L167G/E**_ **in reconstituted airway epithelia developed from the upper (A) and lower (B) respiratory tract and HS/SCARB2 expression in the same tissues (C -D)**. **(A-B)** Viral loads were measured by RT-qPCR 5 dpi in the apically collected washes of the apically or basally inoculated tissues and are expressed as the mean ±SEM (N≥2). ***P<0.001, **P<0.01, *P<0.05. Frequency plots are shown on the right (see Fig 3 for details) **(C-D)** Tissue sections were colored with hematoxylin and eosin (upper panel) or labeled by immunofluorescence. SCARB2 is stained in green, HS in red and cell nuclei in blue. Scale bar = 20 μm.

As only viruses with VP1 97L were detected in the patient bronchoalveolar lavage, the replication of EV71-VP1_97R167G_, EV71-VP1_97L167E_ and EV71-VP1_97R/L167G/E_ was compared in 3D reconstituted human upper (Fig 4A MucilAir) and lower (Fig 4B SmallAir) airway epithelia cultured at an air-liquid interface. These tissues faithfully reproduce the pseudostratified architecture of the upper and lower airway epithelia composed of ciliated cells, mucus secreting cells (as well as club cells in the lower airway) and basal cells (Fig 4C and 4D, upper panels) [40, 41]. Viral suspensions were applied apically (air exposed side) or basally (liquid exposed side) for 4 hours before removal of unbound viruses by extensive washing. Each day afterwards, apically released viruses were collected and basal medium was changed. Viruses released apically at day 5 pi were quantified by RT-qPCR (Fig 4A and 4B). Overall replication was low in both tissue types compared to typical respiratory EVs, such as EV-D68 or rhinoviruses [40, 42], however the infectivity of viruses collected at day 5 pi was confirmed by productive infection in RD cells. No significant difference was observed in the level of replication of EV71-VP1_97R167G_ and EV71-VP1_97L167E_ after apical inoculation of MucilAir (Fig 4A) or SmallAir (Fig 4B). Nevertheless, sequencing of the viral population collected at 5 dpi highlighted that, when inoculated together, EV71-VP1_97L167E_ outcompeted EV71-VP1_97R167G_ (Fig 4A and 4B). In contrast, after basal infection, EV71-VP1_97R167G_ showed a net replication advantage in both individual and competition replication assays.

These results correlate with high expression of HS at the basal tissue side and notably low HS expression at the apical side (Fig 4C and 4D). Interestingly, the expression of SCARB2 showed the opposite pattern with high expression at the apical tissue side and low expression at the basal side (Fig 4C and 4D).

#### Intestinal tissues

**Fig 5:**
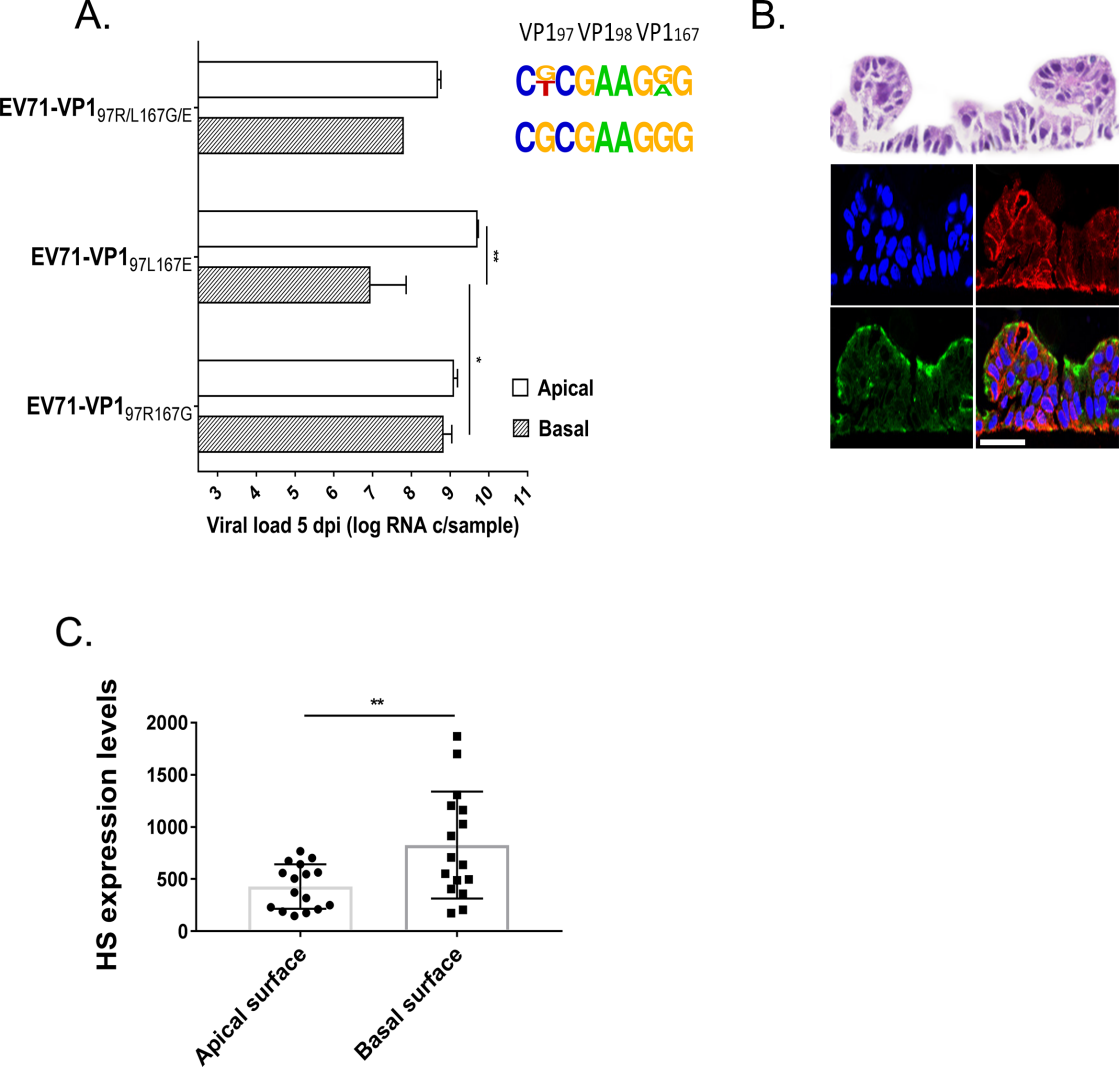
**Replication of EV71-VP1**_**97R167G**_**, EV71-VP1**_**97L167E**_ **and EV71-VP1**_**97R/L167G/E**_ **in reconstituted small intestine tissues (A) and HS/SCARB2 expression in the same tissues (B and C)**. **(A)** Viral loads were measured by RT-qPCR 5 dpi in the apically collected washes of the apically or basally inoculated tissues and are expressed as the mean (±SEM) N = 2. **P<0.01, *P<0.05. The frequency plots are shown on the right (see Fig 3 for details) **(B)** Tissue sections were colored with hematoxylin and eosin (upper panel) or labeled by immunofluorescence. SCARB2 is stained in green, HS in red and cell nuclei in blue. Scale bar = 20 μm. **(C)** Quantification of HS expression at the apical and basal surface of intestinal tissues was achieved through an analysis using MetaMorph software based on the immunofluorescence labeling of HS attachment receptor in sectioned tissues.

As a mixed population of viruses with VP1 97L and VP1 97R was highlighted in the patient’s stool, the experimental approach used for respiratory tissues was applied to assess the replication abilities of EV71-VP1_97R167G_ and EV71-VP1_97L167E_ in 3D reconstituted human small intestine tissues (EpiIntestinal, Mattek) cultured at the air-liquid interface (Fig 5). In this tissue culture model, that reproduces the cellular composition of the small intestine with enterocytes, paneth cells, M cells, tuft cells and intestinal stem cells, viral loads were more than 2 log higher than those measured in respiratory tissues (Fig 5A versus Fig 4A and 4B). For small intestine tissues as for respiratory tissues, there was no significant difference between the replication of EV71-VP1_97R167G_ and EV71-VP1_97L167E_ 5 dpi for the apical inoculation (Fig 5A), while for the basal inoculation, EV71-VP1_97R167G_ replicated significantly better than EV71-VP1_97L167E_ and systematically outcompeted the latter variant in competition. However, in contrast to observations made in respiratory tissues, EV71-VP1_97L167E_ did not outcompete EV71-VP1_97R167G_ after the apical infection, and both populations were present 5 dpi in a mixed viral population. These data are in agreement with the expression of HS in the tissue, with the attachment receptor being expressed both at the apical and basal side of the tissue, although with higher concentration at the basal tissue side (Fig 5B and 5C). Again, for these tissues as for respiratory tissues, SCARB2 expression is more abundant at the apical tissue side (Fig 5B).

#### 2D and 3D engineered neural cultures

**Fig 6:**
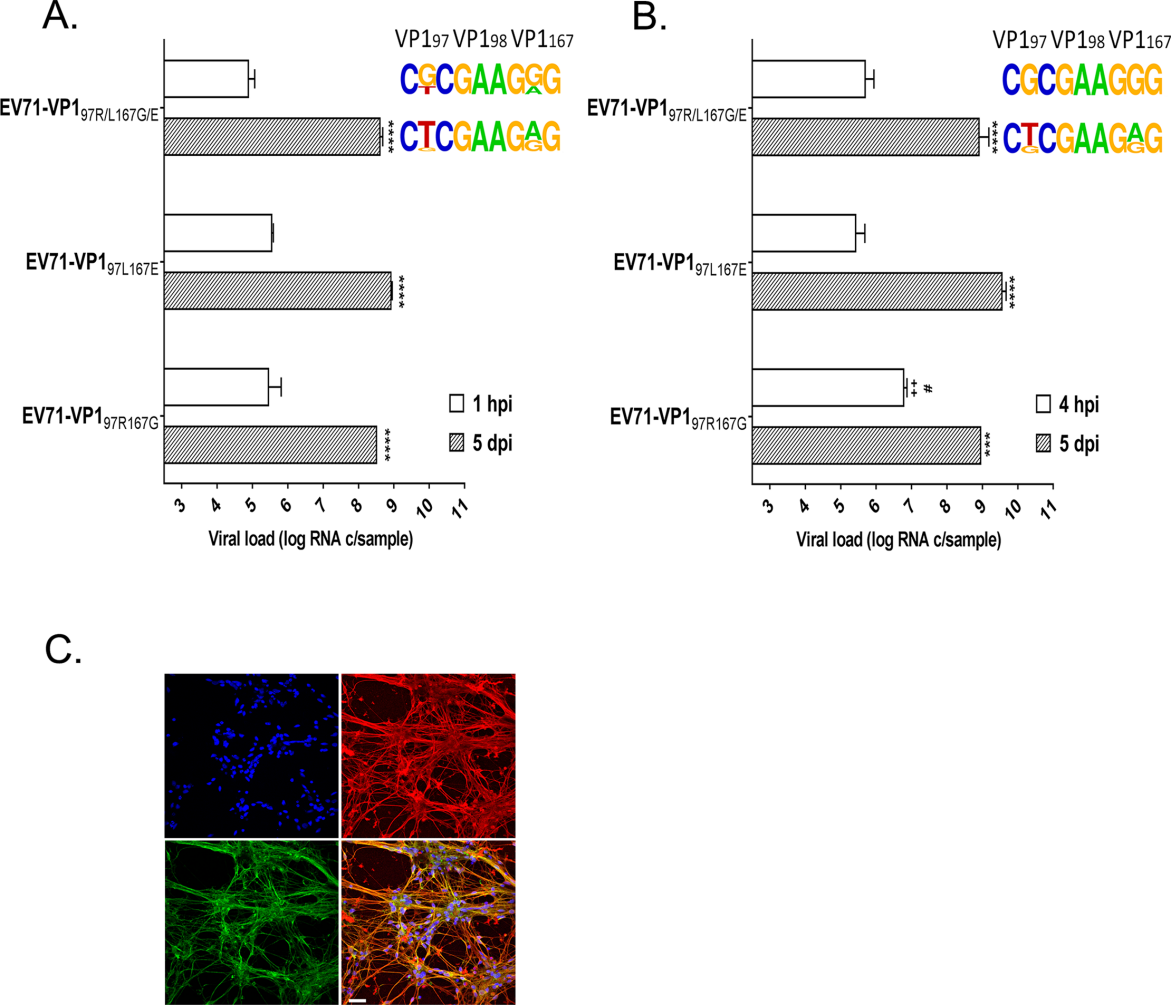
**Replication of EV71-VP1**_**97R167G**_**, EV71-VP1**_**97L167E**_ **and EV71-VP197**_**R/L167G/E**_ **in 2D (A) and 3D (B) neural tissues and HS/SCARB2 expression in the same 2D tissues (C). (A-B)** Viral load was measured by RT-qPCR and expressed as the mean (±SEM). (N = 2). ****P<0.0001, ^++^P<0.01 or ^#^P<0.05. (^+^relative to EV71-VP1_97L167E_ and ^#^ relative to EV71-VP1_97R/L167G/E_ at the same time point). The frequency plots are shown on the right (see Fig 3 for details) **(C)** 2D neural cultures were labeled by immunofluorescence. SCARB2 is stained in green, HS in red and cell nuclei in blue. Scale bar = 20 μm.

**Fig 7:**
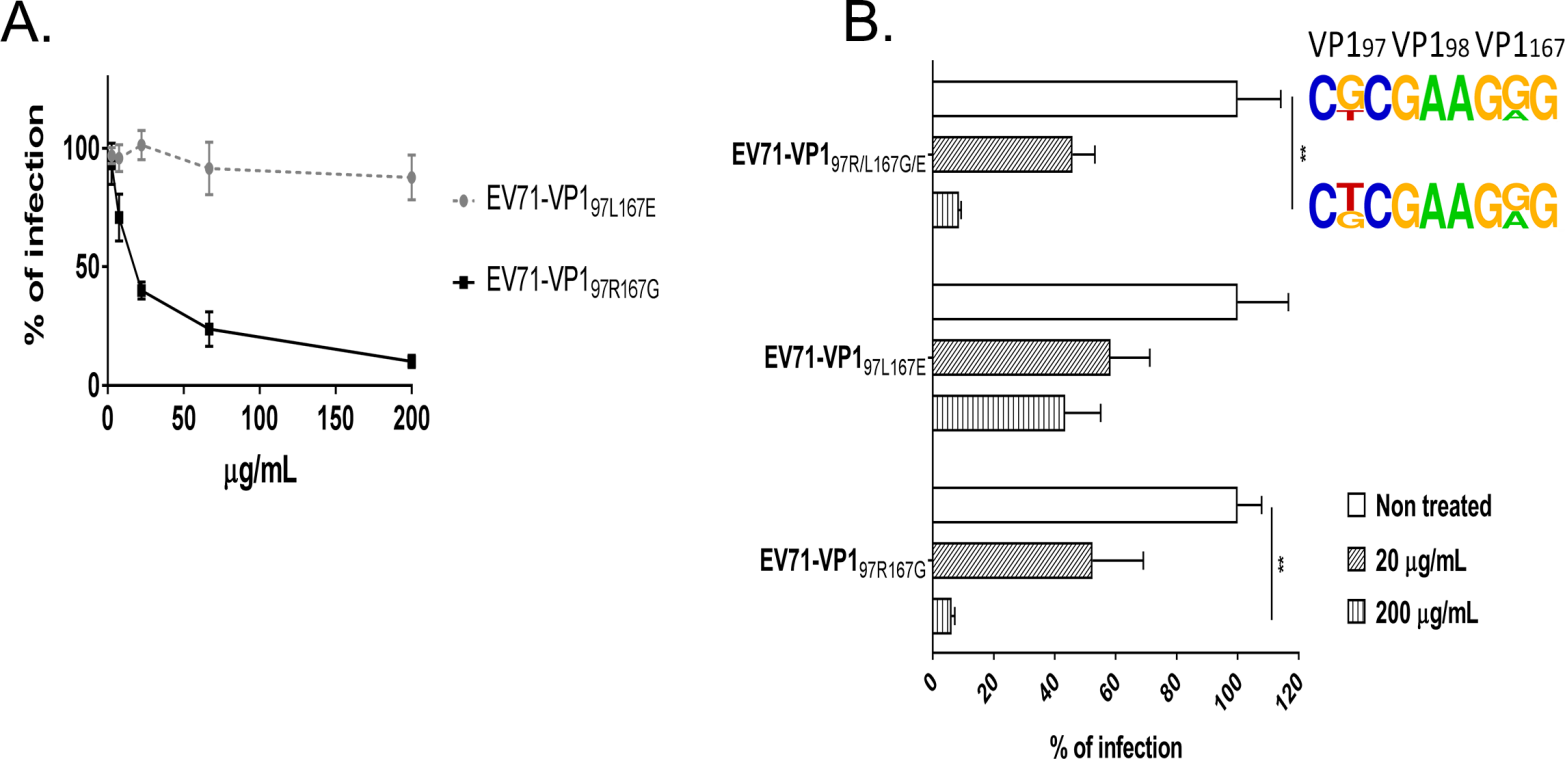
**Viral infectivity of EV71-VP1**_**97R167G**_ **and EV71-VP1**_**97L167E**_ **in Vero cells (A) and of EV71-VP1**_**97R167G**_**, EV71-VP1**_**97L167E**_ **and EV71-VP1**_**97L/R167E/G**_ **in neural cells (B) in the presence of increasing doses of ı-carrageenan.** The percent of infection was measured by an immunocytochemistry assay in Vero cells (A) and by RT-qPCR on cell lysate 1 dpi in neural cells (B) and expressed as the mean (±SEM) compared to non-treated viruses. (N = 2). **P<0.005. The frequency plots are shown on the right (see Fig 3 for details).

Since only EV71 with VP1 97R was sequenced from the patient’s CSF, the replication of the different variants was also compared in engineered 2D (Fig 6A) and 3D (Fig 6B) neural tissues containing a mixed population of mature, functional neurons and glial cells. Viral loads were measured in 2D culture lysates at 1 hpi and 5 dpi and in 3D tissue lysates at 4 hpi and 5 dpi. EV71-VP1_97R167G_, EV71-VP1_97L167E_ and EV71-VP1_97R/L167G/E_ replicated efficiently in both types of cultures and without significant difference 5 dpi. Notably, EV71-VP1_97R167G_ viral loads were significantly higher 4 hpi in 3D tissues. Upon infection with a mixed population, this variant was also dominant at 1 hpi and 4 hpi in 2D and 3D tissues, respectively (Fig 6A and 6B), as well as at 1 dpi in 2D cultures (Fig 7B). However, the replication fitness of EV71-VP1_97L167E_ was greater in competition experiments at 5 dpi. HS expression is abundant in both models, supporting the selection of EV71-VP1_97R167G_ at early time points but not the dominance of EV71-VP1_97L167E_ 5 dpi (Fig 6C). Of note both HS (S2A and S2B Fig) and SCARB2 (S2C and S2D Fig) are highly expressed in neurons and glial cells.

### Iota-carrageenan specifically inhibits infection by EV71-VP1_97R167G_

Because of the acquired ability of EV71-VP1_97R167G_ to bind HS, its sensitivity to iota-carrageenan (ı-carrageenan), a highly sulfated polysaccharide, was investigated and compared to the sensitivity of EV71-VP1_97L167E_ in Vero and 2D neural cultures. As expected, EV71-VP1_97R167G_ infection was strongly inhibited by ı-carrageenan in Vero cells (IC_50_ = 17.8 μg/ml), whereas EV71-VP1_97L167E_ infection was only slightly affected by the treatment (IC_50_>200 μg/ml) (Fig 7A). Similar results were obtained in neural cultures, where the infection of EV71-VP1_97R167G_ and EV71-VP1_97R/L167G/E_ was inhibited up to 90% in a dose-dependent manner. EV71-VP1_97L167E_ infectivity was also inhibited but to a lesser extent (up to 50%). Treatment of the mixed viral population altered the dominant sequence from EV71-VP1_97R167G_ at 1 dpi towards EV71-VP1_97R/L167G/E_ (Fig 7B).

## Discussion

In 2012, we reported an EV71 genogroup C1 disseminated infection in an immunocompromised patient treated with rituximab who was hospitalized with respiratory and neurological symptoms [39]. Although EV71 genogroup C1 infections have not been associated with severe diseases in the past, several complicated cases have been recently reported in Europe, underscoring the importance of defining EV71 virulence determinants [43, 44]. By sequencing the full genome of the virus from different samples, we identified the acquisition of a non-conservative mutation in VP1 (L97R), which was absent at the beginning of the infection in the respiratory tract but was present in a mixed population in the gastrointestinal tract and became dominant in the blood and CSF at later time points. We showed that this mutation conferred a binding advantage to the virus in certain cell lines. In this study, we extended these findings and showed that the VP1 L97R substitution, as well as an associated mutation (VP1 E167G) observed after *in vitro* culturing, confers an ability of the virus to use HS as an attachment receptor while it does not alter the dependence of the virus on the ubiquitously expressed SCARB2 entry receptor. Tan and colleagues recently showed that variants with VP1 E167G do not bind heparin [29]. In addition, in our previous publication, we observed the same binding advantage of the VP1_97R_ mutant in neuroblastoma and Vero cells that was observed in this study with the VP1_97R167G_ mutant [39]. This strongly suggests that the VP1 L97R mutation confers an HS binding ability, while the VP1 E167G mutation has a stabilizing function as proposed previously based on VP1 3D structure [39]. Interestingly, after competition between EV71-VP1_97L167E_ and EV71-VP1_97R167G_ for binding and replication in cells pretreated with heparinase, EV71-VP1_97R_ was excluded from the viral population, while a new variant (EV71-VP1_98K_) which is also known to promote HS binding [29], emerged. Thus, it is very likely that after cleavage with heparinase at the 1→4 linkages between hexosamine and glucuronic acid residues, HS is still bound by EV71-VP1_98K_ but not by EV71-VP1_97R_, suggesting that these two variants present different HS binding specificities.

To assess EV71 replication *in vivo* in the gastrointestinal and respiratory tracts and in the CNS, we analyzed the replication of HS-dependent (EV71-VP1_97R167G_) and independent (EV71-VP1_97L167E_) variants in relevant tissue culture models that mimicked the upper and lower respiratory airway epithelia, intestinal and neural tissues. To reproduce natural infection routes, we inoculated intestinal and respiratory tissue cultures that grow at an air-liquid interface from the apical (corresponding to the lumen) or basal (corresponding to the blood-borne) tissue sides. A comparison of viral growth of HS-dependent and HS-independent variants and competition experiments highlighted that HS-dependent variants had improved access to respiratory and intestinal tissues from the basal tissue side, where HS is highly expressed, while HS-independent variants show improved replication after infection from the air-exposed side of respiratory tissues, where HS is only slightly present, in line with previous publications [45, 46]. Infection from the luminal side of intestinal tissues sustained efficient replication of both variants and correlated with intermediate expression of HS at this side. We also highlighted high expression of SCARB2 at the apical side of respiratory and intestinal tissues and low expression at the basal side. HS may thus be necessary in tissues with low SCARB2 expression to concentrate the virus at the cell surface and promote subsequent interaction with SCARB2. This would explain why HS-dependent variants infect the basal side of respiratory and intestinal tissues better than HS-independent variants.

Our findings in respiratory and intestinal tissues correlate with the viral populations isolated from the different specimens of the patient. We only observed an HS-independent variant in the bronchoalveolar lavage of the patient, a sample collected from the luminal side of the respiratory tissue. Importantly, sequencing of EV71 that was detected in bronchoalveolar lavages from two other immunosuppressed patients who presented neurological complications in Switzerland also identified viruses from the same genogroup and with the same VP1 sequence (GenBank accession numbers MH256663 and MH256664). Of note in these 2 cases as in our case, the infecting virus had a glutamate at position 145, a residue known to confer poor HS binding ability [29]. This suggests that HS-independent variants may be preferentially transmitted via the respiratory route.

We observed a mixed viral population in the stool of the patient, a sample that reflects viral population isolated from the luminal side of the gastrointestinal tract. A mixed viral population was also obtained after apical inoculation of intestinal tissue cultures with a viral population composed of EV71-VP1_97L167E_ and EV71-VP1_97R167G_. Since both *ex vivo* and *in vivo* intestinal tissues are the favored site of EV71 replication, and since these tissues express HS at the basal and apical surface, it is very likely that the emergence of an HS-dependent virus arose at this site. Whether this site was reached after replication in the respiratory tract and propagation via shedding in oral secretions and swallowing or whether infection occurred simultaneously in the two sites remains an open question.

Concerning neurotropism, the viral population sequenced from the CSF of the patient was HS-dependent while results obtained in 2D and 3D neural cultures were more intricate. In single infections, HS-dependent and HS-independent variants revealed similar neurotropic potential as both showed high replication levels in primary neural cells without significant difference 5 dpi. However, in competition experiments, HS-independent variants outcompeted HS-dependent variants 5 dpi while the opposite occurred at earlier time points (1 hpi and 1 dpi in 2D and 4 hpi in 3D cultures). The higher amount of cell-associated HS-dependent viruses shortly after inoculation is easily explicable by the high expression of HS in neural tissues. However, the fact that the improved binding is not reflected by an improved replicative fitness 5 dpi is less obvious. A similar situation was observed in Vero cells where the binding advantage of EV71-VP1_97R167G_ did not result in improved replication relative to EV71-VP1_97L167E_. Whether another step of the viral life is affected by the VP1 L97R mutation in Vero, 2D and 3D neural cultures is currently under investigation. Of note in this study and in our previous publication [39], the HS-dependent variant was fitter in SH-SY5Y neuroblastoma cells at any time post infection. This difference between primary neural cultures and immortalized cells may rely on the nature of these culture models (cancer neuron precursors for neuroblastoma cells versus mature neurons and glial cells for both 2D and 3D neural cultures) and emphasizes the need to use relevant primary tissue culture models to study viral pathogenesis.

Two explanations may account for the detection of HS-dependent virus only in the patient’s CSF and our experimental observations that the HS-independent virus outcompetes the HS-dependent virus in neural tissues 5 dpi (thought both variants replicate to high level in independent infections). First, the patient’s CSF may have been sampled shortly after neural invasion, at a time where the HS-dependent variant was also fitter in neural cultures. However, this would imply that both variants were present together in the CNS where the HS-dependent variants outcompeted the HS-independent variants. The fact that only HS-dependent variants were found in the patient’s blood does not support this hypothesis. More probably, the ability to bind HS promoted EV71-VP1_97R167G_ replication in HS-enriched tissues (such as the basal layers of the gastrointestinal mucosa, muscle or endothelial cells), resulting in sustained viremia and subsequent dissemination to neural tissues. In this context, EV71 neurotropism may not rely on an improved growth in neural tissues but rather on an improved dissemination ability selected upstream of CNS contamination over the course of an infection.

The implication of HS binding in dissemination and neurotropism has been investigated for several viruses and remains controversial. Studies on aphtoviruses [47], flaviviruses [48], togaviruses [49], alphaviruses [50] and even enteroviruses such as coxsackievirus B3 [51] suggest that binding to HS may lower viremia due to virus trapping in tissues expressing high HS levels, while it may improve neurotropism and ability to cross the blood-brain barrier [52]. Of note, two recent papers reported that infection by EV71 with VP1 E145Q substitution was associated with an attenuated phenotype in mice and cynomolgus monkeys and that this attenuation was linked to the ability of VP1 145Q to bind HS [31, 34]. The authors conclude that HS binding and *in vivo* virulence are negatively correlated due to trapping and abortive infection of HS-dependent viruses in tissues expressing high levels of HS but low levels of SCARB2. In the clinical case investigated here, the HS binding variant was the only variant sequenced from the blood of the patient suggesting that HS binding did not prevent viremia and dissemination but rather promoted it. Several hypotheses could explain these different observations: first, mice and monkeys may not faithfully reproduce EV71 infection in human as suggested by studies showing that isolates with VP1 145G/Q are frequently associated with severe neurological disease in humans [35–38]; second, the inoculation site may modulate the infection outcome. Huang and colleagues [53] showed that broad mutant spectra with divergent mutations are observed at the initial infection sites (the respiratory and digestive systems) and that a selection bottleneck occurs afterwards with subsequent enrichment of advantageous mutations in the viral population. Intraperitoneal or intravenous injections may thus prevent the natural evolution of the viral population observed after fecal-oral or respiratory transmission; third, the binding affinity for HS may differ according to the number and/or location of positive charges on the viral capsid, and different mutations (VP1 E145Q versus VP1 L97R) may result in different binding intensities and different trapping strengths; finally, the host immunity certainly plays a key role. In the clinical case presented here, the patient underwent immunosuppressive therapy with rituximab, an anti-CD20 antibody that depletes the peripheral B‐cell pool. Seroneutralisation experiments performed with the patients’ serum at a time when the VP1 97R mutation was already present in the plasma failed to highlight the presence of an antibody-mediated selective immune pressure against HS-dependent and HS-independent variants [39]. This absence of neutralizing antibodies was correlated with a negative complement fixation assay confirming a poor antibody response against enterovirus linked to the immunosuppressed state of the patient. Fujii et al showed that viruses with VP1 145E are more resistant to neutralizing antibodies than viruses with VP1 145G [31]. The absence of immune pressure may thus be a prerequisite for the emergence of HS-dependent variants *in vivo*, since critical residues, such as VP1 98 and 145, seem to play an important role both in immune escape [30, 31] and HS binding [29, 34, 54, 55]. The emergence of HS-dependent mutants and their dissemination could thus be favored in the presence of a poor antibody response. Additional clinical investigations are needed to further assess the implication of HS binding in dissemination and disease severity in humans.

The recent emergence of severe cases associated with EV71 genogroup C1 infections and the lack of an efficient vaccine or antiviral to fight EV71 infections [56, 57] highlights an important need for the development of effective antiviral strategies [58]. In this study, we demonstrated the key role of HS attachment receptor binding in EV71 dissemination in an immunocompromised host, and our preliminary results in cells and neural cultures highlighted the inhibition of HS-dependent variants by soluble HS mimetic compounds. Soluble heparan sulfate analogs may thus be a useful tool to limit enterovirus replication and dissemination. In conclusion, our data may help to unravel the key determinants of EV71 dissemination and neurotropism and bridge the gap between EV71 neurological diseases and the lack of a therapeutic approach.

## Materials and methods

### Ethics statement

Respiratory (http://www.epithelix.com/products/mucilair) and EpiIntestinal (https://www.mattek.com/products/epiintestinal/ tissues) were ordered from Epithelix and MatTek biotechnology companies. There, the tissues are developed from anonymized samples and after Ethical approval. The study was conducted according to the Declaration of Helsinki on biomedical research (Hong Kong amendment, 1989), and the research protocol was approved by our local ethics committee.

### Cells

RD (human rhabdomyosarcoma, ATCC CCL-136) and Vero (monkey kidney, ATCC CRL-1587) cells were cultured in Dulbecco's Modified Eagle Medium (DMEM) and GlutaMAX (31966021, Gibco, Thermo Fisher Scientific, Switzerland) supplemented with 10% (v/v) fetal bovine serum (FBS) (P40-37500, Pan Biotech, Chemie Brunschwig, Switzerland) and 100 μg/ ml of penicillin and streptomycin (15140–122, Gibco, Thermo Fisher Scientific, Switzerland). Caco-2 (human colorectal adenocarcinoma, ATCC HTB 37) and SH-SY5Y cells (human neuroblastoma, ATCC CRL- 2266) were grown in Eagle’s minimum essential medium (EMEM) (BE12-125F, Lonza, Switzerland) and DMEM/F12 medium (31331093, Gibco, Thermo Fisher Scientific, Switzerland), respectively, supplemented with 2 mM L-glutamine (25030–024, Gibco, Thermo Fisher Scientific, Switzerland), 1% non-essential amino acids (11140–035, Gibco, Thermo Fisher Scientific, Switzerland) and 10% (v/v) FBS. HEK 293T/17 (human kidney, ATC C#CRL-11268) cells were grown in DMEM (41965039, Gibco, Thermo Fisher Scientific, Switzerland) supplemented with 10% (v/v) fetal bovine serum (FBS), 100 μg/ ml of penicillin and streptomycin, 2 mM l-glutamine, 1 mM sodium pyruvate (11360–039, Gibco, Thermo Fisher Scientific, Switzerland), and 1% non-essential amino acids.

Cultures were maintained at 37°C in a 5% CO_2_ atmosphere. Infection medium was similar but with reduced FBS concentrations (2.5% for Vero and RD cells and 5% for Caco-2 and SH-SY5Y cells).

### Viral stocks

#### EV71-VP1_97L167E_ and EV71-VP1_97R167G_

The pClVP1_97L167E_ and pClVP1_97R167E_ infectious clones were ordered from Biomatik (Ontario, Canada) as previously described [39]. The VP1 E167G mutation was added by site directed mutagenesis as previously described [59] using 5’GCCAGATTCCAGAGGGTCTCTCGCATGGC3’ forward and 5’GCCATGCGAGAGACCCTCTGGAATCTGGC3’ reverse primers. Constructs were checked by sequencing, linearized with *Bam*HI, *in vitro* transcribed and transfected into Vero or Caco-2 cells, as previously described [39].

Seven days after transfection, supernatants and cells were collected, subjected to 3 freeze-thaw cycles, clarified by centrifugation (5 min at 1000 rpm) and re-passaged 3 times in 80% confluent Vero cells. Alternatively, confluent Vero cells were inoculated with supernatant from infected Caco-2 cells. For the last passage, and when cytopathic effect (CPE) was strong enough, the infection medium was changed 24 h before collection to obtain only freshly produced viruses. Supernatants were collected, clarified by centrifugation (5 min at 1000 rpm) and concentrated by ultracentrifugation as previously described [30]. Ultracentrifuged viral stocks were aliquoted and stored at -80°C. VP1 was PCR amplified and sequenced to confirm the absence of unexpected mutations (see below).

#### EV71-VP1_97R/L167G/E_

Two viral stocks that contained equimolar amounts of EV71-VP1_97R167G_ and EV71-VP1_97L167E_ were used in this study. The first stock was naturally generated by the emergence of spontaneous mutations during viral stock preparation. The presence of equimolar amounts of EV71-VP1_97R167G_ and EV71-VP1_97L167E_ was assessed by Sanger sequencing and was confirmed by subcloning (pCR2.1-TOPO cloning kit, 45–0641, Invitrogen, Thermo Fisher Scientific, Switzerland) and sequencing of VP1 from individual clones. The second stock was created by mixing equimolar amounts of EV71-VP1_97R167G_ and EV71-VP1_97L167E_. Again, equal representation of the two derivatives was assessed by sequencing with the same primers. Both stocks were used in *ex vivo* experiments (in MucilAir, SmallAir, EpiIntestinal and neural tissues) while the naturally generated stock was used for binding and infection assays *in vitro*.

All viral stocks were quantified by RT-qPCR and titration, according to the Reed and Munch method [60] and were passaged less than 3 times to exclude any bias linked to cell adaptation.

### Heparinase assay

Confluent Vero, Caco-2, RD or SH-SY5Y cells pre-plated in a 96 well plate, were washed twice with heparinase III digestion buffer (0.1 M sodium acetate pH 7.0, 1 mM calcium acetate and 0.2% BSA) and incubated for 1 h at 37°C with 3.5 mIU/ml of heparinase III (AMS.HEP_ENZ III_S, Amsbio, Switzerland) (50 μl/well). Control samples were incubated with digestion buffer alone. Heparinase III efficient cleavage was assessed by immunofluorescence with a mouse anti-Δ-heparan sulfate F69-3G10 antibody (370260-S, Amsbio, Switzerland, diluted 1:500) specific for a heparan sulfate neo-epitope generated after digestion.

### Heparin inhibition assay

EV71-VP1_97R/L167G/E_ mixed viral population was pre-incubated with 300 μg/ml of soluble heparin sodium salt from porcine intestinal mucosa (H4784, Sigma-Aldrich, Merck, Switzerland) diluted in culture medium or with culture medium alone as control for 1 h at 37°C. Confluent monolayers of Vero, Caco-2, RD or SH-SY5Y cells were challenged with 50 μl of the pre-incubated virus-heparin or virus-alone mixture and virus binding and replication assays were performed.

### Virus binding and replication assay

Confluent cells were first seeded in 96-well plates and binding and replication assays were performed 24h later as previously described [39]. Briefly, the medium was removed, cells were washed with cold binding buffer (Hanks' Balanced Salt Solution [HBSS, 14175053, Gibco, Thermo Fisher Scientific, Switzerland] containing 1% BSA [wt/vol] and 0.1% sodium azide [wt/vol]) and incubated for 1h on ice with 50 μl of viral suspension containing 5*10^8^ viral RNA copies. After removal of unbound virus with 3 washes with 200 μl of cold binding buffer, cells were either lysed directly in 200 μl of easyMAG lysis buffer to quantify bound viruses by RT-qPCR or overlaid with fresh culture medium and left an additional day at 37°C before lysis and viral load quantification by RT-qPCR. To determine the dominant population in binding and replication experiment run with EV71-VP1_97R/L167G/E_, the VP1 region was PCR-amplified and sequenced.

### Virus binding to immobilized heparin sepharose beads

Aliquots of 200 μl heparin sepharose beads (ab193268, Abcam, UK) were immobilized to Pierce cellulose acetate filter spin cups (69702, Thermo Fisher Scientific, Switzerland) by centrifugation at 1500 rpm for 5 min. The beads were then equilibrated with 600 μl of binding buffer (0.02M Tris-HCl, 0.14M NaCl, pH 7.4) and centrifuged for 5 min at 1500 rpm. Subsequently, 100, 200 or 600 μl of viral suspension of EV71-VP1_97R/L167G/E_ containing respectively 10^9^, 2*10^9^ and 6*10^9^ viral RNA copies were added and incubated with the heparin sepharose beads for 1h at 4°C with gentle mixing. The flow-through fraction was collected by centrifugation (5 min at 1500 rpm) and the heparin sepharose beads were washed 5 times with 200 μl of binding buffer. Bound viral particles were then eluted twice by sequential incubation of 5 min with 200 μl of elution buffer (0.02M Tri-HCl, 2M NaCl, pH 7.4). The collected fractions (input, flow-through and eluate) were analyzed by RT-qPCR and VP1 was PCR amplified and sequenced.

### RNA extraction and real time-quantitative polymerase chain reaction

RNA was extracted using the NucliSens easyMAG magnetic beads system (BioMérieux, France) according to the manufacturer’s instructions. RT-qPCR was performed using the quantitative Entero/Ge/08 assay as previously described [61] in a one-step format using the QuantiTect Probe RT-PCR Kit (Qiagen, Switzerland) according to the manufacturer's instructions in a StepOne Applied Biosystems thermocycler. As a quantitative reference standard for each run, 10-fold dilution series (from 2.5*10^8^ to 2.5*10^5^ copies/ml) of the *in vitro* transcribed full-length pBMH-EV-D68 was used. The RNAse P housekeeping gene (4316831, Thermo Fisher Scientific, Switzerland) was quantified by qPCR in binding and replication experiments to confirm homogeneous cell number.

### RT-PCR and sequencing

Retro-transcription was performed using Superscript II (Invitrogen) and either random hexamer primers (Roche) or a specific primer (2A-3408-R: 5’CTGGGTTTTGAAAAGCTGACC3’) as previously described [39]. The VP1 region was amplified by PCR (Fwd 5’TGCTCGAGATGGAGTATTCG3’ and Rev 5’CTGGGTTTTGAAAAGCTGACC3’) and nested PCR (Fwd 5’CGACTACTACACTACAGGCTTGGTTAG3’ and Rev 5’CATTGGGCGAGGTATCCAC3’) with platinum Taq DNA polymerase (P/N 10966026, Invitrogen, Thermo Fisher Scientific, Switzerland). PCR products were purified as previously described [39] and sequenced (Fasteris-DNA sequencing service, Switzerland).

### Generation of KO cell lines using CRISPR/Cas9

Two SCARB2 targeting guide RNAs (sgRNA1 5’-CACCGCGATGCTGCTTCTACACGGC-3’ & 3’-CGCTACGACGAAGATGTGCCGCAAA-5’, and sgRNA2 5’-CACCGCCGGCATTGTCTGACGTAT-3’ & 3’-CGGCCGTAACAGACTGCATACAAA-5’ for the sgRNA2) were subcloned in the pLentiCRISPRV2 vector (Addgene # 52961). Recombinant lentiviruses (plentiCRISPRV2, pLentiCRISPRV2-SCARB2-sg1 or pLentiCRISPRV2-SCARB2-sg2) were produced by transient transfection of HEK 293T/17 cells as previously described [62]. Caco-2 and RD cells (70–80% confluency in 6-well plates) were transduced with 1 ml of viral supernatants. Five days post transduction the cells were trypsinised and subjected to puromycin selection (58-58-2, Invivogen, LABFORCE, Switzerland; 10 μg/ml for Caco-2 cells and 6 μg/ml for RD cells). Cells were maintained in the selection medium and the expression of SCARB2 was assessed by Western Blot.

### Infection of *in vitro* human reconstituted respiratory (MucilAir, SmallAir) and small intestine tissues (EpiIntestinal)

Human upper (MucilAir) and lower (SmallAir) airway epithelia (Epithelix, Geneva, Switzerland) were developed from isolated nasal polyp and distal lung epithelial cells originating from surgeries as previously described [40, 41]. Human small intestine tissues (EpiIntestinal) were purchased from MatTek (Ashland, USA) and are reconstituted from primary human small intestinal epithelial cells isolated from the ileum of healthy adult donors. After full differentiation, the EpiIntestinal tissues faithfully reproduce the pseudo-stratified architecture of the human small intestine composed of enterocytes, paneth cells, M cells, tuft cells and intestinal stem cells [https://www.mattek.com/products/epiintestinal/].

The tissues, cultured at an air-liquid interface (at 33°C for MucilAir and at 37°C for the other tissues), were infected apically or basally as previously described [63]. Briefly, for apical infection, 10^8^ viral RNA copies (in 100 μl of culture medium) culture medium were applied at the tissue/air interface while for basal infection, 6*10^8^ viral RNA copies (in 600 μl of culture medium) were applied at the tissue/liquid interface. After 4 hours, residual viruses were removed by extensive apical or basal washes and then, every day, 200 μl of culture medium was applied apically for 20 min to collect apically released viruses. Basal medium was also collected daily and replaced with 500 μl of fresh medium. RNA was extracted from apical samples collected 5 dpi.

### Infection of 2D and 3D engineered neural tissues

2D and 3D neural tissues were engineered from human induced pluripotent neural stem cells (GSC-4311, MTI-GlobalStem, Thermo Fisher Scientific, Switzerland) and contained a mixed population of mature neurons and glial cells [64]. 2D cultures were inoculated with 10^7^ viral RNA copies (in 100 μl of culture medium). Residual inoculum was removed after 1 h and tissues were lysed 1 hpi and 5 dpi for RNA extraction. 3D cultures were inoculated with 4*10^7^ viral RNA copies (in 40 μl of medium) and tissues were lysed 4 hpi or 5 dpi for RNA extraction.

For all tissues, replication was quantified by RT-qPCR and VP1 was PCR amplified and sequenced in both single infections or competition assays.

### Western blot and immunofluorescence (IF)

#### Antibodies

Mouse anti-HS (F58-10E4, 370255–1, Amsbio, Switzerland, diluted 1:100 in PBS + 1% BSA), rabbit anti-LIMPII/Igp85 (SCARB2) (PA3-1682, Thermo Fisher Scientific, Switzerland, diluted 1:1000), rabbit anti-GFAP (Z0334, Dako, Agilent, Switzerland, diluted 1:100), mouse anti-GFAP (MAB360, Millipore, Merck, Switzerland, diluted 1:50), rabbit anti-tubulin β-III (Previously Covance Catalog# PRB-435P, Biolegend, Switzerland, diluted 1:1000) and mouse anti-tubulin β-III (T8660, Sigma-Aldrich, Merck, Switzerland, diluted 1:200) were used as primary antibodies for IF staining of respectively HS, SCARB2, glial cells and neurons. Goat anti-mouse Alexa Fluor 594 (A21044, Invitrogen, Thermo Fisher Scientific, Switzerland, diluted 1:2000), goat anti-mouse Alexa Fluor 594 (A11032, Invitrogen, Thermo Fisher Scientific, Switzerland, diluted 1:2000) and goat anti-rabbit Alexa Fluor 488 (A11008, Invitrogen, Thermo Fisher Scientific, Switzerland, diluted 1:2000) were used as secondary antibodies. Rabbit anti- LIMPII/Igp85 (SCARB2) Ab (diluted 1:1000 in 5% milk/TTBS 0.05%), mouse anti-EV71 VP2 mAb (MAB979, Millipore, Merck, Switzerland, diluted 1:1000 in 5% milk/TTBS 0.05%) and mouse anti-GAPDH mAb (6C5, sc-32233, Santa Cruz, Switzerland, diluted 1:1000 in 5% milk/TTBS 0.05%) were used as primary antibodies for western blot while anti-rabbit HRP-labelled Ab (7074, Cell Signaling Technology; diluted 1:1000 in 5% milk/TTBS 0.05%) or anti-mouse HRP-labelled Ab (7076, Cell Signaling Technology; diluted 1:1000 in 5% milk/TTBS 0.05%) were used as secondary antibodies.

### Western blot

Proteins were loaded on a 10% SDS-PAGE gel, transferred on a PVDF membrane (162–0177, BIO-RAD, Switzerland) that was hybridized with the primary Ab overnight at 4°C. The membranes were washed twice with TTBS 0.05% [TBS buffer (10 mM Tris HCl, pH 7.5, 500 mM NaCl) with 0.05% Tween] for 10 min and incubated for 1 h at 37°C with the secondary Ab. The membranes were washed twice with TTBS for 10 min and developed with the ECL system (34080, Thermo Fisher Scientific, Switzerland) according to the manufacturer’s protocol. Images were acquired with the Fujifilm LAS 4000 luminescence imager.

#### IF on cells

Cells plated on coverslips were washed twice with phosphate-buffered saline (PBS) and fixed for 20 min at room temperature (RT) in a 4% paraformaldehyde solution. Fixed cells were washed with PBS before incubation for 1 h at 37°C with the primary Ab. Intensive PBS washings were performed prior to incubation for 45 min at 37°C with the secondary Ab. Cells were then washed 3 times with PBS, stained with 4,6-diamidino-2-phenylindole (DAPI) for 7 min at RT and washed with PBS a final time. Coverslips were mounted in Fluoroprep mounting medium (BioMérieux, France)

#### IF on paraffin-embedded tissue sections

Paraffin was removed from MucilAir, SmallAir and EpiIntestinal tissue sections by embedding the sections twice in UltraClear solution (3905.5000PE, Biosystems, Switzerland) for 3 min and twice in EtOH 96% (E/0600DF/15, Fisher Chemical, Thermo Fisher Scientific, Switzerland) for 3 min. Sections were then air-dried for 5 min and quickly immersed in water. Tissues were rinsed with PBS, permeabilized with 1% Triton (Fluka, Switzerland) for 15 min at RT and incubated with primary antibodies at 4°C overnight. Intensive PBS washes were performed prior to incubation with the secondary antibody for 45 min at 37°C. Next, tissues were washed 3 times with PBS, stained with DAPI at RT for 7 min, and washed with PBS a final time. Coverslips were mounted in Fluoroprep mounting medium (BioMérieux, France). Alternatively, tissue sections were counterstained with Mayer’s Hematoxylin Solution (5 min), eosin (7 min) and mounted with Néo Mount medium (1.09016.0500, Merck, Switzerland). Images were acquired with a Zeiss LSM 700 Meta confocal microscope with the "Plan-Neofluar" 63x/1.4 oil objective and were processed with the ZEN Blue Advanced Processing and Analysis module.

#### IF on 2D neural cultures

2D neural cultures were fixed at RT in 4% PFA for 20 min, rinsed with PBS permeabilized with 0.1% Triton for 15 min at RT, and embedded in blocking buffer (PBS-BSA 10%) before being incubated with primary antibodies at 4°C overnight. The cultures were washed with PBS prior to incubation with the secondary antibody for 1 hour at RT. Goat anti-mouse Alexa Fluor 594 and goat anti-rabbit Alexa Fluor 488 were used as secondary Ab. After PBS washes, the nuclei were stained with DAPI at RT for 7 min and washed again with PBS. Coverslips were mounted in Fluoroprep mounting medium (BioMérieux, France). All IF Images were acquired with a Zeiss LSM 700 Meta confocal microscope and were processed with the ZEN Blue Advanced Processing and Analysis module.

### MetaMorph analysis in cell lines and intestinal tissues

Images were acquired on a Zeiss LSM700 confocal microscope with a 63x/1.4 oil objective leading to the calibration of 0.396 microns per pixel. HS expression was measured with MetaMorph software version 7.7.6 (Molecular Devices, Sunnyvale, CA).

#### Cell lines

Blue (DAPI) and red (HS) or green (SCARB2) color channels were separated with the ColorSeparate function, and both images were converted to compatible files. A cell scoring step followed, in which the blue channel was used in “All nuclei” section and the red or green channel was used as a positive marker with the following respective parameters: “Approximate min width” (7 and 1 microns), “Approximate max width” (17 and 12 microns), “Parameter Intensity above local background” (8 and 40 levels). The “Algorithm” parameter was set to Standard. The cell scoring step reported the “Total Cells” number to ensure the statistical reliability of measurements, and the parameter “%Positive Cells” to reflect the expression of the receptor.

#### Intestinal tissues

Images were acquired as stacks in which four channels/wavelengths were present (blue: 440 nm; red: 645 nm; green: 510 nm; and transmission light) and four to six z positions were acquired. Red channel planes were first selected with the Select Plane function. Two regions of exactly the same size were manually drawn in the image that corresponded to apical and basal areas of the tissue and both areas were measured and stored. Next, we looped through z planes of the red channel and performed the following actions for each of the two regions: “Area of the intensity above level 3” was measured as Total Tissue Area; “Area of the intensity above level 15” was measured as High Intensity Tissue Area; and values of the “High Intensity Tissue Area” across all z planes were averaged, with values being obtained for the apical and basal areas.

### I-carrageenan inhibition assay

I-carrageenan (C1138, Sigma-Aldrich, Merck, Switzerland) was serially diluted and added for 1h at 37°C to EV71-VP1_97R167G_, EV71-VP1_97L167E_ or EV71-VP1_97R/L167E/G_ (multiplicity of infection [MOI], 0.01 PFU/cell). The mixture was then added to confluent 2D neural cells or Vero cells grown in a 96-well plate at a density of 13 × 10^4^/well. After 1 h at 37°C, monolayers were washed and overlaid with fresh medium. One dpi, viral replication was monitored by immunocytochemistry (ICC) in Vero cells and by RT-qPCR in neural cells.

For ICC, cells were washed with PBS, fixed with cold methanol:acetone (1:1) for 1 min and permeabilised with PBS-Triton 0.1% for 5 min on ice. Cells were then incubated 1h at 37°C with mouse anti-EV71 VP2 monoclonal antibody (MAB979, Millipore, Merck, Switzerland; diluted 1:500 in PBS-BSA 1%). After 3 washes with PBS-Triton 0.1%, cells were incubated 1 h at 37°C with the anti-mouse HRP-labelled secondary antibody (7076, Cell Signaling Technology; diluted 1:1000 in PBS-BSA 1%). After extensive washing, DAB substrate solution (D4293-50SET, Sigma-Aldrich, Merck, Switzerland) was added for 15 min before final washing with PBS. More than 400 cells per well were scored under a light microscope. The percent of inhibition of virus infectivity was determined by comparing the percentage of infected cells in presence of increasing concentrations of ı-carrageenan relative to the percent of infected cells in absence of ı-carrageenan (set as reference at 100%). All data were generated from duplicate wells in three independent experiments.

For quantification by RT-qPCR, neural cultures were lysed in 200 μl of easyMAG lysis buffer. RNA was extracted, viral replication was quantified by RT-qPCR and VP1 was PCR amplified and sequenced. The percent of inhibition of virus infectivity was determined by comparing the viral load measured in neural cells in presence of increasing concentrations of ı-carrageenan relative to the viral load measured in absence of ı-carrageenan (set as reference at 100%).

### Statistics

Values are expressed as mean (± SEM). Experiments were performed at least in biological duplicates, with N in the figure legend indicating the number of replicates. Two-way ANOVA, Fisher’s exact tests and determination of the fifty percent inhibitory concentration (IC_50_) was determined using GraphPad Prism 7.02 software.

## Supporting information

S1 Fig Human SCARB2 is necessary for successful infection by EV71-VP1_97R167G_, EV71-VP1_97L167E_ variants in mouse cells. Mouse L929 cells were transfected with a control plasmid (pCTL) or a plasmid expressing human SCARB2 (pSCARB2). 24h post transfection, cells were infected with EV71-VP1_97R167G_ or EV71-VP1_97L167E_ variants at a MOI = 0.2. 24 h later, cells were lysed and the expression of SCARB2 and of EV71 VP0 and VP2 proteins were quantified by western blot. Of note, the antibody used to detect SCARB2 is not specific for human SCARB2 and recognises endogenous mouse SCARB2.
(TIF)

S2 Fig Glial cells and mature neurons express HS and SCARB2. Co-localisation of HS expressing cells (stained in red) with **(A)** glial cells (stained in green) and **(B)** neurons (stained in green). Co-localisation of SCARB2 expressing cells (stained in green) with **(C)** glial cells (stained in red) or **(D)** neurons (stained in red). For each panel subpanel showing cell nuclei and merged images are also shown. Scale bar = 20 μm.
(TIF)

S1 Text Exogenous expression of hSCARB2 in mouse cells (L929). The pCWX-UBI-SCARB2-PGK-GFP SCARB2 expressing vector was constructed with the Gateway cloning technology according to the manufacturer’s instruction (Gateway LR Clonase II Enzyme mix, 11791020, Invitrogen, Thermo Fisher Scientific, Switzerland). An LR recombination reaction was performed between the entry clone containing the SCARB2 coding sequence (pENTR-L1-SCARB2-L2, Clone ID IOH9776, Invitrogen, Thermo Fisher Scientific, Switzerland), the entry clone containing the ubiquitin promoter (pENTR-L4-UBI-L1R, gift from Patrick Salmon, Addgene plasmid # 45959) and the destination vector containing attR sites (pCWX-R4-DEST-R2-PGK-GFP, kindly provided by Prof. Karl-Heinz Krause, University of Geneva). Sub confluent mouse L929 cells, were transfected with pCWX-UBI-SCARB2-PGK-GFP or pCLX-UBI-GFP (gift from Patrick Salmon, Addgene # 27245). After 24 h, the cells were infected with EV71-VP1_97R167G_ and EV71-VP1_97L167E_ at an MOI of 0.2. 24 h post infection cells were lysed with RIPA (Tris 50 mM- pH 7, NaCl 150 mM, 0.1% SDS, 0.5% Sodium deoxycholate, 1% Triton X-100) and analysed by Western Blot both for quantification of SCARB2 expression and to highlight expression of viral protein. To this end, protein were loaded on a 10% SDS-PAGE gel, transferred on a PVDF membrane (162–0177, BIO-RAD, Switzerland) that was hybridized with the primary rabbit anti- LIMPII/Igp85 (SCARB2) Ab (PA3-1682, Thermo Fisher Scientific, Switzerland, diluted 1:1000 in 5% milk/TTBS 0.05%), mouse anti-EV71 VP2 mAb (MAB979, Millipore, Merck, Switzerland, diluted 1:1000 in 5% milk/TTBS 0.05%) and mouse anti-GAPDH mAb (6C5, sc-32233, Santa Cruz, Switzerland) overnight at 4°C. The membranes were incubated for 1 h at 37°C with the anti-rabbit HRP-labelled secondary antibody (7074, Cell Signaling Technology; diluted 1:1000 in 5% milk/TTBS 0.05%) or the anti-mouse HRP-labelled secondary antibody (7076, Cell Signaling Technology; diluted 1:1000 in 5% milk/TTBS 0.05%).
(DOCX)
