# Supplementary figures and images for: A VP1 mutation acquired during an enterovirus 71 disseminated infection confers heparan sulfate binding ability and modulates ex vivo tropism
Source: PLoS Pathog. 2018 Aug 3;14(8):e1007190. doi: 10.1371/journal.ppat.1007190 (PMC6093697; doi:10.1371/journal.ppat.1007190)

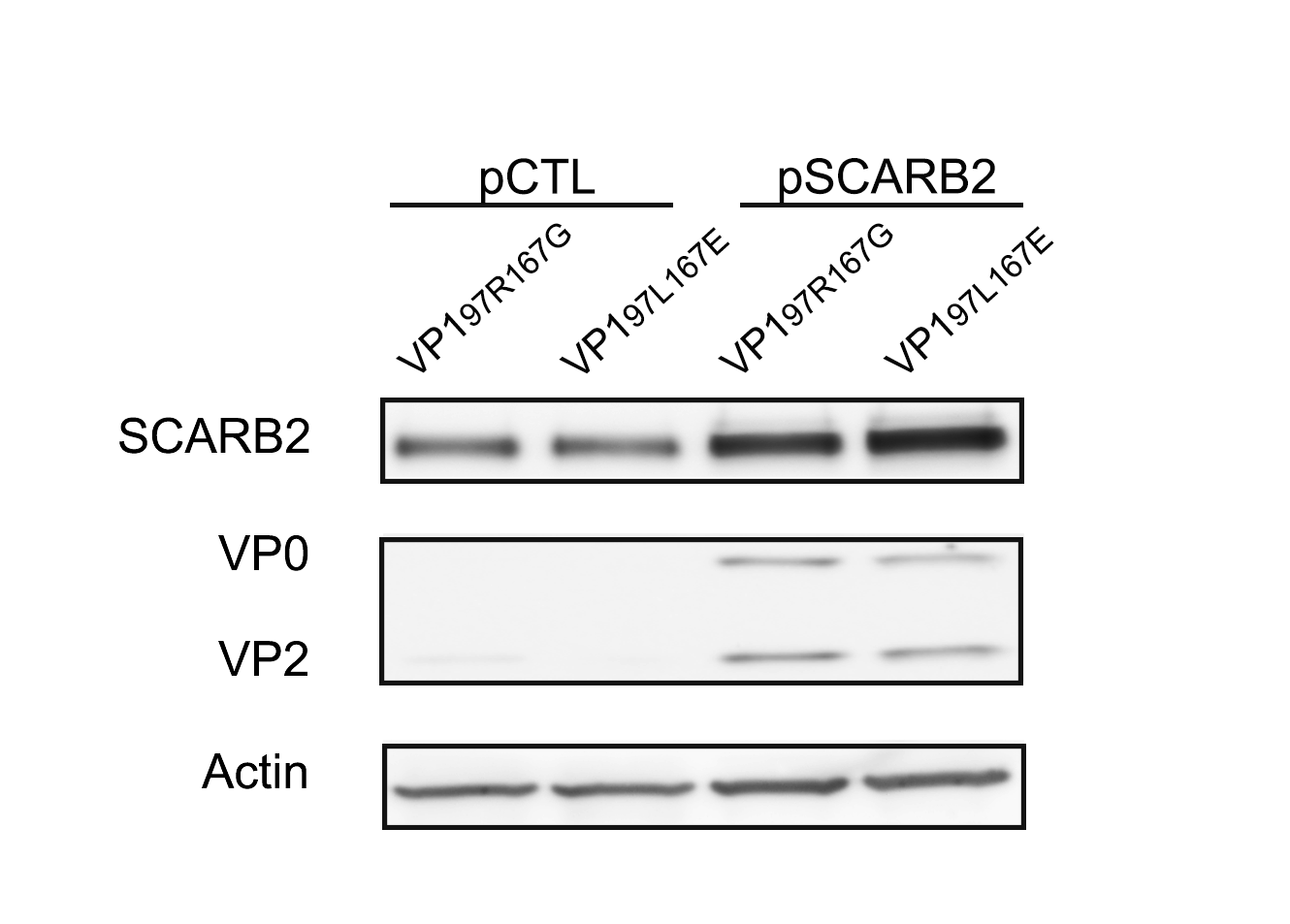

Supplement: S1 Fig — Mouse L929 cells were transfected with a control plasmid (pCTL) or a plasmid expressing human SCARB2 (pSCARB2). 24h post transfection, cells were infected with EV71-VP197R167G or EV71-VP197L167E variants at a MOI = 0.2. 24 h later, cells were lysed and the expression of SCARB2 and of EV71 VP0 and VP2 proteins were quantified by western blot. Of note, the antibody used to detect SCARB2 is not specific for human SCARB2 and recognises endogenous mouse SCARB2. (TIF) [file ppat.1007190.s001.tif]

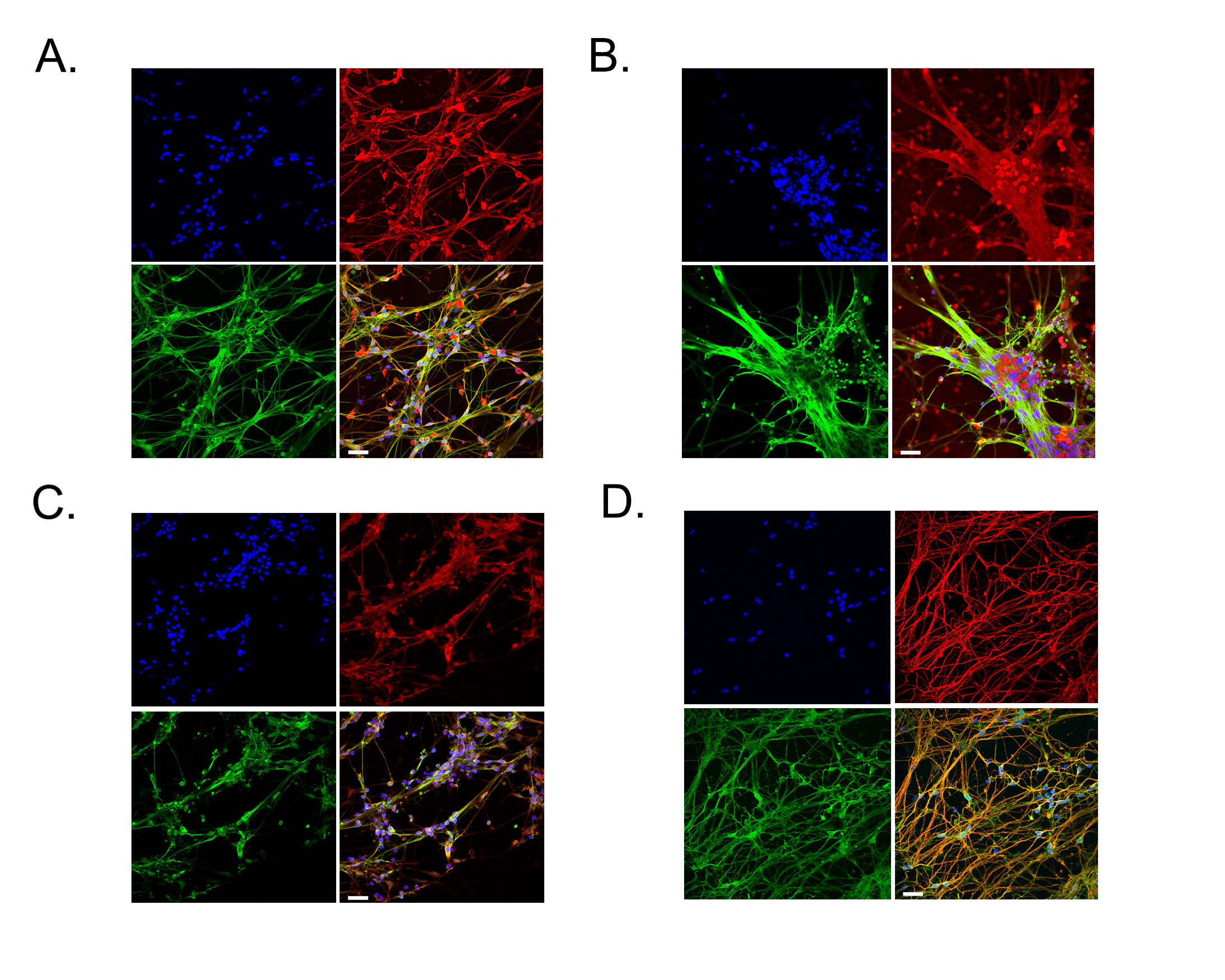

Supplement: S2 Fig — Co-localisation of HS expressing cells (stained in red) with (A) glial cells (stained in green) and (B) neurons (stained in green). Co-localisation of SCARB2 expressing cells (stained in green) with (C) glial cells (stained in red) or (D) neurons (stained in red). For each panel subpanel showing cell nuclei and merged images are also shown. Scale bar = 20 μm. (TIF) [file ppat.1007190.s002.tif]
