# Supplementary material for: A VP1 mutation acquired during an enterovirus 71 disseminated infection confers heparan sulfate binding ability and modulates ex vivo tropism
Source: PLoS Pathog. 2018 Aug 3;14(8):e1007190. doi: 10.1371/journal.ppat.1007190 (PMC6093697; doi:10.1371/journal.ppat.1007190)
Supplement: S1 Text — The pCWX-UBI-SCARB2-PGK-GFP SCARB2 expressing vector was constructed with the Gateway cloning technology according to the manufacturer’s instruction (Gateway LR Clonase II Enzyme mix, 11791020, Invitrogen, Thermo Fisher Scientific, Switzerland). An LR recombination reaction was performed between the entry clone containing the SCARB2 coding sequence (pENTR-L1-SCARB2-L2, Clone ID IOH9776, Invitrogen, Thermo Fisher Scientific, Switzerland), the entry clone containing the ubiquitin promoter (pENTR-L4-UBI-L1R, gift from Patrick Salmon, Addgene plasmid # 45959) and the destination vector containing attR sites (pCWX-R4-DEST-R2-PGK-GFP, kindly provided by Prof. Karl-Heinz Krause, University of Geneva). Sub confluent mouse L929 cells, were transfected with pCWX-UBI-SCARB2-PGK-GFP or pCLX-UBI-GFP (gift from Patrick Salmon, Addgene # 27245). After 24 h, the cells were infected with EV71-VP197R167G and EV71-VP197L167E at an MOI of 0.2. 24 h post infection cells were lysed with RIPA (Tris 50 mM- pH 7, NaCl 150 mM, 0.1% SDS, 0.5% Sodium deoxycholate, 1% Triton X-100) and analysed by Western Blot both for quantification of SCARB2 expression and to highlight expression of viral protein. To this end, protein were loaded on a 10% SDS-PAGE gel, transferred on a PVDF membrane (162–0177, BIO-RAD, Switzerland) that was hybridized with the primary rabbit anti- LIMPII/Igp85 (SCARB2) Ab (PA3-1682, Thermo Fisher Scientific, Switzerland, diluted 1:1000 in 5% milk/TTBS 0.05%), mouse anti-EV71 VP2 mAb (MAB979, Millipore, Merck, Switzerland, diluted 1:1000 in 5% milk/TTBS 0.05%) and mouse anti-GAPDH mAb (6C5, sc-32233, Santa Cruz, Switzerland) overnight at 4°C. The membranes were incubated for 1 h at 37°C with the anti-rabbit HRP-labelled secondary antibody (7074, Cell Signaling Technology; diluted 1:1000 in 5% milk/TTBS 0.05%) or the anti-mouse HRP-labelled secondary antibody (7076, Cell Signaling Technology; diluted 1:1000 in 5% milk/TTBS 0.05%). (DOCX) [file ppat.1007190.s003.docx]

**S1 Text. Exogenous expression of hSCARB2 in mouse cells (L929)**

The pCWX-UBI-SCARB2-PGK-GFP SCARB2 expressing vector was constructed with the Gateway cloning technology according to the manufacturer’s instruction (Gateway LR Clonase II Enzyme mix, 11791020, Invitrogen, Thermo Fisher Scientific, Switzerland). An LR recombination reaction was performed between the entry clone containing the SCARB2 coding sequence (pENTR-L1-SCARB2-L2, Clone ID IOH9776, Invitrogen, Thermo Fisher Scientific, Switzerland), the entry clone containing the ubiquitin promoter (pENTR-L4-UBI-L1R, gift from Patrick Salmon, Addgene plasmid # 45959) and the destination vector containing attR sites (pCWX-R4-DEST-R2-PGK-GFP, kindly provided by Prof. Karl-Heinz Krause, University of Geneva). Sub confluent mouse L929 cells, were transfected with pCWX-UBI-SCARB2-PGK-GFP or pCLX-UBI-GFP (gift from Patrick Salmon, Addgene # 27245). After 24 h, the cells were infected with EV71-VP1_97R167G_ and EV71-VP1_97L167E_ at an MOI of 0.2. 24 h post infection cells were lysed with RIPA (Tris 50 mM- pH 7, NaCl 150 mM, 0.1% SDS, 0.5% Sodium deoxycholate, 1% Triton X-100) and analysed by Western Blot both for quantification of SCARB2 expression and to highlight expression of viral protein. To this end, protein were loaded on a 10% SDS-PAGE gel, transferred on a PVDF membrane (162-0177, BIO-RAD, Switzerland) that was hybridized with the primary rabbit anti- LIMPII/Igp85 (SCARB2) Ab (PA3-1682, Thermo Fisher Scientific, Switzerland, diluted 1:1000 in 5% milk/TTBS 0.05%), mouse anti-EV71 VP2 mAb (MAB979, Millipore, Merck, Switzerland, diluted 1:1000 in 5% milk/TTBS 0.05%) and mouse anti-GAPDH mAb (6C5, sc-32233, Santa Cruz, Switzerland) overnight at 4°C. The membranes were incubated for 1 h at 37°C with the anti-rabbit HRP-labelled secondary antibody (7074, Cell Signaling Technology; diluted 1:1000 in 5% milk/TTBS 0.05%) or the anti-mouse HRP-labelled secondary antibody (7076, Cell Signaling Technology; diluted 1:1000 in 5% milk/TTBS 0.05%).
